# Dimethyl­bis­(pyrazine-2-carboxyl­ato-κ^2^ *N* ^1^,*O*)tin(IV)

**DOI:** 10.1107/S160053681204295X

**Published:** 2012-10-20

**Authors:** Marzieh Vafaee, Ezzatollah Najafi, Mostafa M. Amini, Seik Weng Ng

**Affiliations:** a Department of Chemistry, General Campus, Shahid Beheshti University, Tehran 1983963113, Iran; b Department of Chemistry, University of Malaya, 50603 Kuala Lumpur, Malaysia

## Abstract

In the title compound, [Sn(CH_3_)_2_(C_5_H_3_N_2_O_2_)_2_], the Sn^IV^ atom is twice *N*,*O*-chelated by two pyrazine-2-carboxyl­ate ligands. The distorted six-coordination is completed by two tin-bound methyl C atoms. The C_2_N_2_O_2_ donor set defines a skewed trapezoidal–bipyramidal geometry. Inter­molecular π–π inter­actions between the pyrazine rings [centroid–centroid distance = 3.8112 (13) Å] are observed.

## Related literature

For background to organotin compounds, see: Dakternieks *et al.* (2003 ▶); Tiekink (1991 ▶); Yin *et al.* (2005 ▶).
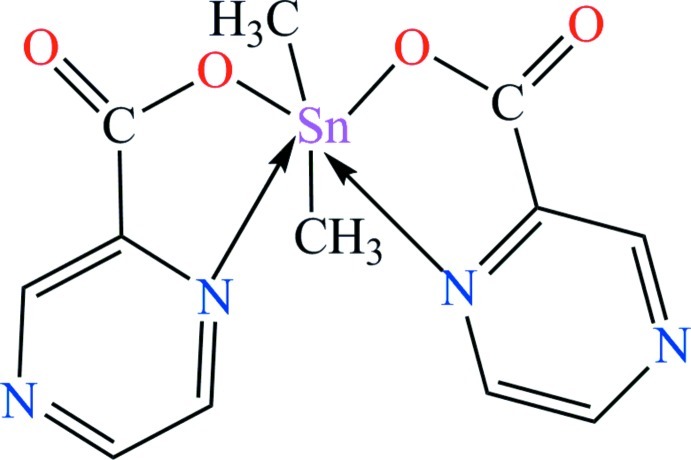


## Experimental

### 

#### Crystal data

[Sn(CH_3_)_2_(C_5_H_3_N_2_O_2_)_2_]
*M* *_r_* = 394.95
Monoclinic, 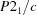

*a* = 9.2887 (6) Å
*b* = 12.3253 (7) Å
*c* = 12.6596 (7) Å
β = 103.738 (1)°
*V* = 1407.88 (14) Å^3^
*Z* = 4
Mo *K*α radiation
μ = 1.84 mm^−1^
*T* = 295 K
0.40 × 0.40 × 0.20 mm

#### Data collection

Bruker APEXII CCD diffractometer
Absorption correction: multi-scan (*SADABS*; Sheldrick, 1996 ▶) *T*_min_ = 0.527, *T*_max_ = 0.710
8657 measured reflections
3193 independent reflections
2892 reflections with *I* > 2σ(*I*)
*R*_int_ = 0.022

#### Refinement

*R*[*F* ^2^ > 2σ(*F* ^2^)] = 0.020
*wR*(*F* ^2^) = 0.051
*S* = 1.06
3193 reflections
192 parameters
H-atom parameters constrained
Δρ_max_ = 0.35 e Å^−3^
Δρ_min_ = −0.47 e Å^−3^

### 

Data collection: *APEX2* (Bruker, 2007 ▶); cell refinement: *SAINT* (Bruker, 2007 ▶); data reduction: *SAINT*; program(s) used to solve structure: *SHELXS97* (Sheldrick, 2008 ▶); program(s) used to refine structure: *SHELXL97* (Sheldrick, 2008 ▶); molecular graphics: *X-SEED* (Barbour, 2001 ▶); software used to prepare material for publication: *publCIF* (Westrip, 2010 ▶).

## Supplementary Material

Crystal structure: contains datablock(s) global, I. DOI: 10.1107/S160053681204295X/hy2594sup1.cif

Structure factors: contains datablock(s) I. DOI: 10.1107/S160053681204295X/hy2594Isup2.hkl

Additional supplementary materials:  crystallographic information; 3D view; checkCIF report

## Figures and Tables

**Table 1:** Selected bond lengths (Å)

Sn1—C1	2.097 (2)
Sn1—C2	2.095 (2)
Sn1—O1	2.1506 (16)
Sn1—O3	2.1238 (15)
Sn1—N1	2.6646 (18)
Sn1—N3	2.5107 (18)

**Fig. 1.**
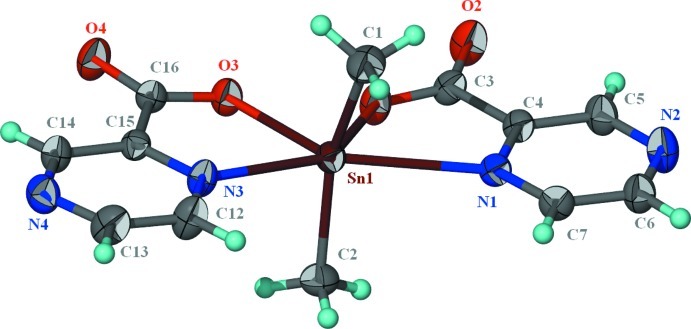
Molecular structure of the title compound. Displacement ellipsoids are drawn at the 50% probability level.

## supplementary crystallographic information

### Comment

The reaction of appropriate amount of dimethyltin dichloride and pyrazine-2-carboxylic acid in dry methanol provided the title compound in good yield. The six-coordinated geometry around the Sn^IV^ atom is better described as skew-trapezoidal bipyramidal (Fig. 1, Table 1). Intermolecular π–π interactions between the pyrazine rings [centroid–centroid distance = 3.8112 (13) Å] stabilize the structure.

### Experimental

Dimethyltin dichloride (0.22 g, 1 mmol) was treated with sodium methoxide (0.1 g, 2 mmol) in methanol (10 ml) to produce dimethyltin dimethoxide and sodium chloride. The sodium chloride precipitate was removed by filtration and then pyrazine-2-carboxylic acid (0.248 g, 2 mmol) in methanol (20 ml) was added to the filtrate. The solution was refluxed for 3 h. Evaporation of the solvent yielded a white solid, which was purified by recrystallization from methanol (yield: 70%. m.p.: 118–120°C).

### Refinement

H atoms were placed in calculated positions and refined as riding atoms, with C–H = 0.93 (CH) and 0.96 (CH_3_) Å and with *U*_iso_(H) = 1.2(1.5 for methyl)*U*_eq_(C). Omitted reflections owing to bad disagreement were (-9 10 6), (-6 13 3), (8 0 4), (2 0 8) and (-1 8 6).

### Crystal data

**Table:**

[Sn(CH_3_)_2_(C_5_H_3_N_2_O_2_)_2_]	*F*(000) = 776
*M**_r_* = 394.95	*D*_x_ = 1.863 Mg m^−^^3^
Monoclinic, *P*2_1_/*c*	Mo *K*α radiation, λ = 0.71073 Å
Hall symbol: -P 2ybc	Cell parameters from 5890 reflections
*a* = 9.2887 (6) Å	θ = 2.3–28.3°
*b* = 12.3253 (7) Å	µ = 1.84 mm^−^^1^
*c* = 12.6596 (7) Å	*T* = 295 K
β = 103.738 (1)°	Prism, colorless
*V* = 1407.88 (14) Å^3^	0.40 × 0.40 × 0.20 mm
*Z* = 4	

### Data collection

**Table:**

Bruker APEXII CCD diffractometer	3193 independent reflections
Radiation source: fine-focus sealed tube	2892 reflections with *I* > 2σ(*I*)
Graphite monochromator	*R*_int_ = 0.022
φ and ω scans	θ_max_ = 27.5°, θ_min_ = 2.3°
Absorption correction: multi-scan (*SADABS*; Sheldrick, 1996)	*h* = −12→11
*T*_min_ = 0.527, *T*_max_ = 0.710	*k* = −16→13
8657 measured reflections	*l* = −15→16

### Refinement

**Table:**

Refinement on *F*^2^	Primary atom site location: structure-invariant direct methods
Least-squares matrix: full	Secondary atom site location: difference Fourier map
*R*[*F*^2^ > 2σ(*F*^2^)] = 0.020	Hydrogen site location: inferred from neighbouring sites
*wR*(*F*^2^) = 0.051	H-atom parameters constrained
*S* = 1.06	*w* = 1/[σ^2^(*F*_o_^2^) + (0.0201*P*)^2^ + 0.7349*P*] where *P* = (*F*_o_^2^ + 2*F*_c_^2^)/3
3193 reflections	(Δ/σ)_max_ = 0.001
192 parameters	Δρ_max_ = 0.35 e Å^−^^3^
0 restraints	Δρ_min_ = −0.47 e Å^−^^3^

### Fractional atomic coordinates and isotropic or equivalent isotropic displacement parameters (Å^2^)

**Table:**

	*x*	*y*	*z*	*U*_iso_*/*U*_eq_	
Sn1	0.826212 (14)	0.699389 (11)	0.602141 (11)	0.02943 (6)	
O1	0.87907 (17)	0.71196 (14)	0.44616 (13)	0.0415 (4)	
O2	0.99121 (19)	0.78275 (16)	0.32619 (13)	0.0493 (4)	
O3	0.62830 (16)	0.65997 (14)	0.48535 (12)	0.0374 (3)	
O4	0.39196 (17)	0.61066 (15)	0.43503 (13)	0.0469 (4)	
N1	1.09601 (19)	0.77904 (14)	0.61292 (14)	0.0320 (4)	
N2	1.3488 (2)	0.87890 (18)	0.57415 (17)	0.0478 (5)	
N3	0.62453 (19)	0.65022 (15)	0.69497 (14)	0.0340 (4)	
N4	0.3759 (2)	0.57674 (16)	0.76062 (16)	0.0421 (4)	
C1	0.7815 (3)	0.86219 (19)	0.6305 (2)	0.0466 (6)	
H1A	0.8346	0.9084	0.5918	0.070*	
H1B	0.8123	0.8772	0.7070	0.070*	
H1C	0.6770	0.8755	0.6057	0.070*	
C2	0.9233 (3)	0.54697 (19)	0.6433 (2)	0.0444 (5)	
H2A	1.0075	0.5391	0.6123	0.067*	
H2B	0.8523	0.4912	0.6155	0.067*	
H2C	0.9546	0.5408	0.7210	0.067*	
C3	0.9857 (2)	0.76167 (19)	0.41998 (17)	0.0346 (4)	
C4	1.1116 (2)	0.79754 (16)	0.51203 (17)	0.0302 (4)	
C5	1.2359 (3)	0.8484 (2)	0.49323 (19)	0.0405 (5)	
H5	1.2409	0.8618	0.4219	0.049*	
C6	1.3319 (3)	0.86057 (19)	0.67419 (19)	0.0417 (5)	
H6	1.4074	0.8812	0.7331	0.050*	
C7	1.2068 (3)	0.81211 (17)	0.69396 (18)	0.0361 (5)	
H7	1.1996	0.8023	0.7654	0.043*	
C12	0.6207 (3)	0.6469 (2)	0.79961 (18)	0.0445 (6)	
H12	0.7030	0.6696	0.8523	0.053*	
C13	0.4970 (3)	0.6103 (2)	0.83094 (19)	0.0449 (6)	
H13	0.4986	0.6092	0.9047	0.054*	
C14	0.3798 (2)	0.58104 (18)	0.65644 (18)	0.0367 (5)	
H14	0.2974	0.5577	0.6042	0.044*	
C15	0.5024 (2)	0.61905 (16)	0.62266 (16)	0.0295 (4)	
C16	0.5045 (2)	0.62962 (16)	0.50495 (17)	0.0321 (4)	

### Atomic displacement parameters (Å^2^)

**Table:**

	*U*^11^	*U*^22^	*U*^33^	*U*^12^	*U*^13^	*U*^23^
Sn1	0.02845 (8)	0.03258 (9)	0.02919 (9)	0.00030 (5)	0.01068 (6)	0.00025 (5)
O1	0.0333 (8)	0.0637 (11)	0.0291 (8)	−0.0101 (7)	0.0106 (6)	−0.0007 (7)
O2	0.0415 (9)	0.0830 (13)	0.0266 (8)	−0.0074 (8)	0.0144 (7)	0.0015 (8)
O3	0.0337 (8)	0.0518 (9)	0.0280 (7)	−0.0071 (7)	0.0097 (6)	0.0015 (7)
O4	0.0346 (8)	0.0667 (11)	0.0359 (8)	−0.0095 (8)	0.0012 (7)	−0.0012 (8)
N1	0.0310 (9)	0.0379 (9)	0.0296 (9)	0.0011 (7)	0.0123 (7)	0.0020 (7)
N2	0.0421 (11)	0.0589 (13)	0.0449 (11)	−0.0164 (9)	0.0151 (9)	−0.0052 (10)
N3	0.0278 (8)	0.0442 (10)	0.0309 (9)	−0.0032 (7)	0.0088 (7)	−0.0047 (8)
N4	0.0380 (10)	0.0471 (11)	0.0464 (11)	−0.0019 (8)	0.0203 (9)	0.0023 (9)
C1	0.0429 (13)	0.0383 (12)	0.0571 (15)	0.0043 (10)	0.0089 (11)	−0.0073 (11)
C2	0.0437 (13)	0.0380 (12)	0.0511 (14)	0.0060 (10)	0.0104 (11)	0.0047 (10)
C3	0.0303 (10)	0.0456 (12)	0.0303 (10)	0.0039 (9)	0.0119 (8)	−0.0010 (9)
C4	0.0287 (10)	0.0363 (10)	0.0289 (10)	0.0026 (8)	0.0134 (8)	−0.0002 (8)
C5	0.0397 (12)	0.0510 (13)	0.0353 (11)	−0.0075 (10)	0.0175 (9)	0.0000 (10)
C6	0.0394 (12)	0.0449 (13)	0.0404 (12)	−0.0090 (10)	0.0082 (10)	−0.0043 (10)
C7	0.0402 (12)	0.0396 (11)	0.0296 (11)	0.0002 (9)	0.0106 (9)	−0.0006 (8)
C12	0.0348 (11)	0.0687 (16)	0.0308 (11)	−0.0037 (11)	0.0093 (9)	−0.0065 (11)
C13	0.0424 (12)	0.0627 (15)	0.0338 (11)	0.0054 (11)	0.0171 (10)	0.0010 (11)
C14	0.0301 (10)	0.0415 (12)	0.0399 (12)	−0.0040 (9)	0.0109 (9)	−0.0025 (9)
C15	0.0276 (9)	0.0286 (10)	0.0333 (10)	0.0016 (7)	0.0091 (8)	−0.0016 (8)
C16	0.0305 (10)	0.0330 (10)	0.0328 (10)	−0.0004 (8)	0.0079 (8)	−0.0006 (8)

### Geometric parameters (Å, º)

**Table:**

Sn1—C1	2.097 (2)	C1—H1A	0.9600
Sn1—C2	2.095 (2)	C1—H1B	0.9600
Sn1—O1	2.1506 (16)	C1—H1C	0.9600
Sn1—O3	2.1238 (15)	C2—H2A	0.9600
Sn1—N1	2.6646 (18)	C2—H2B	0.9600
Sn1—N3	2.5107 (18)	C2—H2C	0.9600
O1—C3	1.274 (3)	C3—C4	1.508 (3)
O2—C3	1.228 (3)	C4—C5	1.383 (3)
O3—C16	1.288 (3)	C5—H5	0.9300
O4—C16	1.221 (2)	C6—C7	1.382 (3)
N1—C7	1.332 (3)	C6—H6	0.9300
N1—C4	1.339 (3)	C7—H7	0.9300
N2—C6	1.332 (3)	C12—C13	1.378 (3)
N2—C5	1.334 (3)	C12—H12	0.9300
N3—C12	1.334 (3)	C13—H13	0.9300
N3—C15	1.334 (3)	C14—C15	1.389 (3)
N4—C13	1.324 (3)	C14—H14	0.9300
N4—C14	1.329 (3)	C15—C16	1.501 (3)
			
C2—Sn1—C1	154.98 (10)	Sn1—C2—H2C	109.5
C2—Sn1—O3	102.62 (8)	H2A—C2—H2C	109.5
C1—Sn1—O3	99.44 (8)	H2B—C2—H2C	109.5
C2—Sn1—O1	96.58 (8)	O2—C3—O1	124.5 (2)
C1—Sn1—O1	100.72 (9)	O2—C3—C4	118.9 (2)
O3—Sn1—O1	74.09 (6)	O1—C3—C4	116.63 (18)
C2—Sn1—N3	89.54 (8)	N1—C4—C5	121.7 (2)
C1—Sn1—N3	87.17 (9)	N1—C4—C3	116.59 (18)
O3—Sn1—N3	69.63 (6)	C5—C4—C3	121.71 (19)
O1—Sn1—N3	143.67 (6)	N2—C5—C4	122.1 (2)
C2—Sn1—N1	88.49 (8)	N2—C5—H5	118.9
C1—Sn1—N1	82.01 (8)	C4—C5—H5	118.9
O3—Sn1—N1	140.17 (5)	N2—C6—C7	122.7 (2)
O1—Sn1—N1	66.64 (6)	N2—C6—H6	118.7
N3—Sn1—N1	149.56 (5)	C7—C6—H6	118.7
C3—O1—Sn1	129.40 (14)	N1—C7—C6	121.4 (2)
C16—O3—Sn1	126.66 (13)	N1—C7—H7	119.3
C7—N1—C4	116.35 (18)	C6—C7—H7	119.3
C7—N1—Sn1	134.17 (14)	N3—C12—C13	121.1 (2)
C4—N1—Sn1	109.12 (13)	N3—C12—H12	119.5
C6—N2—C5	115.7 (2)	C13—C12—H12	119.5
C12—N3—C15	116.99 (19)	N4—C13—C12	122.8 (2)
C12—N3—Sn1	132.12 (15)	N4—C13—H13	118.6
C15—N3—Sn1	110.85 (13)	C12—C13—H13	118.6
C13—N4—C14	115.86 (19)	N4—C14—C15	122.4 (2)
Sn1—C1—H1A	109.5	N4—C14—H14	118.8
Sn1—C1—H1B	109.5	C15—C14—H14	118.8
H1A—C1—H1B	109.5	N3—C15—C14	120.76 (19)
Sn1—C1—H1C	109.5	N3—C15—C16	116.52 (18)
H1A—C1—H1C	109.5	C14—C15—C16	122.70 (18)
H1B—C1—H1C	109.5	O4—C16—O3	124.5 (2)
Sn1—C2—H2A	109.5	O4—C16—C15	119.47 (19)
Sn1—C2—H2B	109.5	O3—C16—C15	116.07 (17)
H2A—C2—H2B	109.5		
			
C2—Sn1—O1—C3	−98.1 (2)	C7—N1—C4—C5	−0.1 (3)
C1—Sn1—O1—C3	63.8 (2)	Sn1—N1—C4—C5	173.91 (17)
O3—Sn1—O1—C3	160.6 (2)	C7—N1—C4—C3	−179.20 (18)
N3—Sn1—O1—C3	163.69 (17)	Sn1—N1—C4—C3	−5.2 (2)
N1—Sn1—O1—C3	−12.59 (18)	O2—C3—C4—N1	175.7 (2)
C2—Sn1—O3—C16	83.58 (19)	O1—C3—C4—N1	−3.5 (3)
C1—Sn1—O3—C16	−84.53 (19)	O2—C3—C4—C5	−3.4 (3)
O1—Sn1—O3—C16	176.92 (19)	O1—C3—C4—C5	177.4 (2)
N3—Sn1—O3—C16	−1.15 (17)	C6—N2—C5—C4	−2.2 (4)
N1—Sn1—O3—C16	−173.35 (15)	N1—C4—C5—N2	2.0 (4)
C2—Sn1—N1—C7	−81.4 (2)	C3—C4—C5—N2	−178.9 (2)
C1—Sn1—N1—C7	75.4 (2)	C5—N2—C6—C7	0.7 (4)
O3—Sn1—N1—C7	170.55 (17)	C4—N1—C7—C6	−1.4 (3)
O1—Sn1—N1—C7	−179.2 (2)	Sn1—N1—C7—C6	−173.53 (16)
N3—Sn1—N1—C7	5.1 (3)	N2—C6—C7—N1	1.2 (4)
C2—Sn1—N1—C4	106.05 (15)	C15—N3—C12—C13	1.5 (4)
C1—Sn1—N1—C4	−97.16 (15)	Sn1—N3—C12—C13	−175.99 (18)
O3—Sn1—N1—C4	−1.98 (18)	C14—N4—C13—C12	−0.5 (4)
O1—Sn1—N1—C4	8.22 (13)	N3—C12—C13—N4	0.0 (4)
N3—Sn1—N1—C4	−167.44 (13)	C13—N4—C14—C15	−0.5 (3)
C2—Sn1—N3—C12	77.7 (2)	C12—N3—C15—C14	−2.4 (3)
C1—Sn1—N3—C12	−77.5 (2)	Sn1—N3—C15—C14	175.58 (16)
O3—Sn1—N3—C12	−178.7 (2)	C12—N3—C15—C16	176.3 (2)
O1—Sn1—N3—C12	178.2 (2)	Sn1—N3—C15—C16	−5.6 (2)
N1—Sn1—N3—C12	−8.5 (3)	N4—C14—C15—N3	2.0 (3)
C2—Sn1—N3—C15	−99.93 (15)	N4—C14—C15—C16	−176.7 (2)
C1—Sn1—N3—C15	104.88 (15)	Sn1—O3—C16—O4	177.83 (17)
O3—Sn1—N3—C15	3.72 (13)	Sn1—O3—C16—C15	−1.4 (3)
O1—Sn1—N3—C15	0.6 (2)	N3—C15—C16—O4	−174.2 (2)
N1—Sn1—N3—C15	173.84 (12)	C14—C15—C16—O4	4.6 (3)
Sn1—O1—C3—O2	−164.70 (18)	N3—C15—C16—O3	5.1 (3)
Sn1—O1—C3—C4	14.5 (3)	C14—C15—C16—O3	−176.11 (19)
